# Editorial: Multi-omics approaches in disease microbiology: from biomarkers to therapeutic interventions

**DOI:** 10.3389/fmicb.2025.1767864

**Published:** 2026-01-12

**Authors:** Chandrajit Lahiri, Debashis Banerjee, Datta Madamwar, Chirajyoti Deb

**Affiliations:** 1 Department of Biotechnology, Atmiya University, Rajkot, India; 2 P.D. Patel Institute of Applied Sciences (PDPIAS), Charotar University of Science and Technology (CHARUSAT), Anand, India; 3 Translational Research Lab, Arnold Palmer Hospital for Children, Orlando, FL, United States

**Keywords:** diagnostic innovation, disease microbiology, host-microbe interaction, multi-omics integration, systems biology

In recent years, disease microbiology has evolved through the integration of multi-omics approaches. Combining genomic, transcriptomic, proteomic, metabolomic and microbiome analyses now allows researchers to capture the emergence and evolution of infections and their responses to therapy. This transformation is not only technological but also conceptual, reflecting a growing understanding that pathogens, hosts, and environments are interconnected systems rather than isolated entities (Hasin et al., 2017; Chetty and Blekhman, 2024). By capturing data across multiple biological layers, these approaches provide the resolution needed to understand complex infectious processes—where microbial signals, host responses, and environmental cues intersect. This broader shift is reflected in the nine articles gathered under this Research Topic. Together, they show how multi-omics can reveal new disease mechanisms, identify biomarkers, improve diagnostic accuracy, and expand our understanding of ecological and population-level interactions.

Some studies in this Research Topic illustrate how multi-omics approaches are reshaping our understanding of pathogenesis (Zhang et al.; Guo et al.). In one investigation of *Candida albicans*, researchers examined the delicate balance between benign gut colonization and invasive infection. By combining microbiome sequencing, metabolite profiling, and host transcriptomics, they uncovered the molecular and microbial cues that shift this balance—reduced PD-1 signaling, depleted short-chain fatty acids, and loss of beneficial taxa such as *Dubosiella* (Zhang et al.). This study serves as a reminder that infection risk depends not only on the presence of a microbe but also on the surrounding host and metabolic environment. Furthermore, the work on retinopathy of prematurity, where integration of 16S rRNA sequencing with metabolomics revealed temporal changes in the infant gut associated with disease onset, reflects this integrative viewpoint (Guo et al.). Characteristic microbial shifts—including an increase in genera such as *Klebsiella* and *Staphylococcus* alongside reduced *Bifidobacterium* abundance—were accompanied by alterations in metabolites linked to fatty-acid and steroid-hormone biosynthesis. Together, these findings highlight how disruptions in early gut ecology and metabolism may contribute to the disease's progression and offer a basis for potential diagnostic biomarkers.

Other contributions emphasize the growing clinical impact of diagnostic innovation. For instance, compared with conventional tests, third-generation nanopore sequencing markedly improved sensitivity and specificity for extrapulmonary tuberculosis, identifying cases that would otherwise have gone undetected (Song et al.). In another study, metagenomic next-generation sequencing of bronchoalveolar lavage fluid from immunocompromised patients almost doubled pathogen detection rates relative to culture and serology, leading to timely treatment adjustments and improved outcomes (Xin et al.). Together, these findings reveal a changing diagnostic arena—one that is becoming faster, more inclusive, and less constrained by predefined expectations. Complementing these advances are reviews that bridge research innovation with clinical application. The discussion on MALDI-TOF mass spectrometry traces how this once-specialized laboratory tool evolved into a routine instrument for rapid microbial identification, antimicrobial resistance profiling, and even genetic analysis (Xiong and Guan). It also calls attention to the growing need for database expansion and standardization as this technology continues to mature. Looking toward the future, a computational study on multi-omics-guided image classification explores how molecular and visual data can be integrated to develop diagnostic models that are both interpretable and adaptive (Lin et al.). Together, these articles showcase a diagnostic paradigm that is steadily shifting from descriptive testing to intelligent, data-driven discovery. In doing so, these clinical advances illustrate how technology and translational insight are converging to redefine diagnostic precision and patient management.

Beyond individual applications, this Research Topic highlights the importance of cross-domain integration—connecting mechanistic insights, diagnostic advances, and ecological perspectives to reveal how microbial dynamics unfold within and beyond clinical boundaries. In an insightful study of *Hyalomma dromedarii* ticks collected from Tunisia and Saudi Arabia, researchers found that geography and sex influence the tick microbiome's structure and stability (Kratou et al.). Tunisian ticks, dominated by *Francisella* endosymbionts, displayed more resilient microbial networks, suggesting adaptation to arid environments. Such observations expand our understanding of disease vectors, illustrating how environmental pressures shape microbial ecosystems and, in turn, pathogen transmission potential.

Another study brings the focus back to humans and the intergenerational echoes of infection. Infants born to mothers who contracted COVID-19 during pregnancy showed lower microbial diversity and greater variability in their gut microbiomes, regardless of the trimester in which infection occurred (Ignatyeva et al.). These findings suggest that maternal infections can alter early microbial colonization patterns, potentially influencing immune development. The work underscores how microbial disruptions during pregnancy may leave subtle yet lasting imprints on infant health—an area that remains vital for post-pandemic research and surveillance.

Threaded through these contributions is the recognition that integration, not just innovation, defines the future of disease microbiology. The review on sepsis exemplifies this idea by showing how multi-omics can untangle the complex host–microbiome interactions that drive this life-threatening condition (Lu et al.). The authors outline strategies for combining heterogeneous data and highlight the challenges that remain—ranging from computational harmonization to clinical interpretability. Yet, their discussion also points to opportunity: as analytical tools grow more refined, multi-omics may enable truly personalized and adaptive approaches to infection management.

Taken together, the nine articles in this Research Topic capture a field in active transformation. They demonstrate multi-omics evolving from fragmented observation to integrated understanding—connecting mechanisms with diagnostics, ecology with epidemiology, and computational analysis with clinical practice. The picture that emerges is one of microbiology becoming not only descriptive but decisively actionable. By linking molecular depth with system-level awareness, these studies chart a path toward a future in which infection biology is understood as a continuum that spans from the microbe's niche to the patient's bedside, and from data to decision (Zhang et al., 2010). In that sense, this Research Topic stands as both a snapshot of current progress and a preview of where integrative microbiology is heading.
